# Prognostic Impact and Clinical Features of Spread through Air Spaces in Operated Lung Cancer: Real-World Analysis

**DOI:** 10.3390/medicina60081374

**Published:** 2024-08-22

**Authors:** Sedat Yildirim, Ozkan Alan, Zeynep Yuksel Yasar, Tugba Kaya, Goncagul Akdag, Oguzcan Kinikoglu, Gonca Gul Gecmen, Alper Yasar, Deniz Isik, Heves Surmeli, Tugba Basoglu, Ozlem Nuray Sever, Mahmut Emre Yildirim, Hatice Odabas, Nedim Turan

**Affiliations:** 1 Department of Medical Oncology, Health Science University, Kartal Dr. Lütfi Kirdar City Hospital, Istanbul 34865, Turkey; dr_zeynepyuksel@hotmail.com (Z.Y.Y.); tugbakaya89@hotmail.com (T.K.); akdaggoncagul@gmail.com (G.A.); ogokinikoglu@yahoo.com (O.K.); alperyasar1@gmail.com (A.Y.); dnz.1984@yahoo.com (D.I.); hevessurmeli@hotmail.com (H.S.); basoglutugba@gmail.com (T.B.); ozlem.sever@hotmail.com (O.N.S.); emremahmutyildirim@gmail.com (M.E.Y.); odabashatice@yahoo.com (H.O.); turan.nedim@hotmail.com (N.T.); 2 Division of Medical Oncology, School of Medicine, Koc University, Istanbul 34450, Turkey; ozkan.alan@hotmail.com; 3 Department of Pathology, Health Science University, Kartal Dr. Lütfi Kirdar City Hospital, Istanbul 34865, Turkey; gonca.gecmen@hotmail.com

**Keywords:** disease-free survival, non-small-cell lung cancer, overall survival, prognostic factor, tumor spread through air spaces

## Abstract

*Background and Objectives*: Lung cancer is the leading cause of cancer-related deaths. Spread through air spaces (STAS) is an adverse prognostic factor that has become increasingly known in recent years. This study aims to investigate the impact of STAS presence on overall survival (OS) and disease-free survival (DFS) in patients with surgically resected stage IA-IIIA lung cancer and to identify clinicopathological features associated with STAS. *Materials and Methods*: This research involved 311 lung cancer surgery patients. The relationship between the presence of STAS in the patients’ surgical pathology and OS and DFS values was examined. Clinicopathological features associated with the presence of STAS were determined. *Results*: There were 103 (33%) STAS-positive patients. Adenocarcinoma histological subtype, perineural invasion (PNI), and lymphovascular invasion (LVI) were significantly correlated with being STAS positive. STAS significantly predicted DFS and OS. One-year and five-year DFS rates were significantly lower in the STAS-positive group compared to the STAS-negative group (65% vs. 88%, 29% vs. 62%, respectively, *p* ≤ 0.001). Similarly, one-year and five-year OS rates were significantly lower in the STAS-positive group compared to the STAS-negative group (92% vs. 94%, 54% vs. 88%, respectively, *p* ≤ 0.001). In multivariate analysis, STAS was found to be an independent prognostic factor for both DFS and OS (HR: 3.2 (95%CI: 2.1–4.8) and 3.1 (95%CI: 1.7–5.5), *p* < 0.001 and <0.001, respectively). *Conclusions*: In our study, STAS was found to be an independent prognostic biomarker in operated stage IA-IIIA lung cancer patients. It may be a beneficial pathological biomarker in predicting the survival of patients and managing their treatments.

## 1. Introduction

Lung cancer continues to be the leading cause of cancer-related deaths in both women and men worldwide and is a global public health problem [1,2]. Non-small-cell lung cancer (NSCLC) has several histological subtypes, including adenocarcinoma, squamous cell carcinoma, and large-cell carcinoma, and adenocarcinoma is the most common subtype [3,4]. While half of the patients are in stage 1, 2, and operable stage 3 at the time of diagnosis, approximately 40% are in the metastatic stage, and 5-year overall survival (OS) decreases as the stage of the disease progresses [4,5]. Currently, surgical removal is linked to improved survival rates in cases with early-stage illness. Hence, it is essential to accurately determine which patients are appropriate candidates for surgery upon diagnosis and to subsequently provide adjuvant therapy, if determined to be required, after the surgical procedure [4,6].

Surgical resection remains the mainstay of treatment for non-small-cell lung cancer (NSCLC) up to stage 3A, even with recent advancements in treatment options. Platinum-based chemotherapy is the preferred treatment for patients with resectable stage IB-IIIA NSCLC as an adjuvant therapy. It is considered the most effective and important part of current treatment methods [7]. While adjuvant chemotherapy has positively impacted overall survival (OS), recurrence rates continue to pose a significant challenge [8,9]. Various characteristics have been identified as negative risk factors for overall survival (OS) and disease-free survival (DFS), including tumor differentiation, wedge resection, vascular invasion, visceral pleural invasion, and unclear lymph node status [10]. Additionally, research has shown that tumor markers like CEA, SUV uptake value on PET CT, poor performance status (PS), tumor spread through air spaces (STAS), and molecular parameters such as the Ki-67 and KRAS status can influence OS and DFS [9].

Apart from known risk factors, environmental factors, medication side effects, and the patient’s daily lifestyle can affect the patient’s survival [11,12,13]. Environmental variables may influence survival as well as contribute to the disease’s development [11]. It has been proposed that the rising occurrence of lung adenocarcinoma could be attributed to other variables besides smoking, including the impact of environmental factors on biological pathways and energy metabolism, as well as hypoxia and oxidative stress. These impacts have the potential to modify the tumor microenvironment and impact the prognosis of lung cancer as well as the cellular response to the illness [11,14,15]. It may also lead to drug interactions and affect prognosis. Furthermore, as our understanding of aspects like hypoxia in cancer improves, it may potentially provide valuable insights for the identification of novel medicines in cancer therapy via these pathways [16]. Recently, researchers have examined novel pathological characteristics and biomarkers in lung cancer, as well as other types of cancer, to determine their potential predictive value. One of the important predictive biomarkers for progression and survival in non-advanced lung cancer is STAS, which is obtained through tumor histology and whose importance is becoming increasingly evident [16,17].

STAS is a new prognostic factor that has been identified in many lung cancer subtypes, especially lung adenocarcinoma [18]. It was described by the World Health Organization in 2015 as an invasion pattern showing tumor foci spreading through air spaces at the border of the primary tumor [19]. It is estimated that 15–60% of STAS lung cancer patients are positive [20]. From a clinical and pathological perspective, the presence of STAS is more often seen in individuals who have lymphatic and pleural invasion, poorly differentiated tumors bigger than 1 cm, a history of smoking, and advanced stages of the disease [21]. In addition, recurrence rates are higher in STAS-positive patients, and it is a negative independent prognostic factor in terms of OS and DFS [22]. However, studies show that it does not predict recurrence and has no prognostic value on OS and DFS [23,24].

In our study, we aimed to investigate the impact of STAS presence on overall survival (OS) and disease-free survival (DFS) in patients with surgically resected stage IA-IIIA lung cancer and to identify clinicopathological features associated with STAS.

## 2. Materials and Methods

### 2.1. Study Population

This research comprised patients who underwent surgery and follow-up care at Kartal Doctor Lütif Kırdar City Hospital Medical Oncology Clinic after receiving a lung cancer diagnosis between 1 October 2016 and 1 October 2023. The restaging procedure was conducted in accordance with the IASLC Lung Cancer Staging Project Edition of the TNM 8 Classification for Lung Cancer [4]. This research comprised patients who had non-metastatic resectable stage I–III illness and underwent surgery, with or without neoadjuvant treatment. Patients who had metastatic cancer at the time of diagnosis, a second malignancy that was currently active, showed progression despite receiving neoadjuvant treatment, were deemed inoperable, were under the age of 18, or had not undergone evaluation of their STAS status in the surgical pathology report for any reason were not included in the study.

### 2.2. Data Collection

The age, gender, smoking status, histological features of the tumor, and the recurrence and survival status of the patients were gathered by retrospectively reviewing their medical records. STAS is a method of lung neoplasm invasion that has recently been identified and assessed using hematoxylin and eosin (H&E) staining [25]. STAS refers to the invasion of air gaps in the lung parenchyma by micro-papillary clusters, solid nests, or solitary cells, extending beyond the initial tumor [26,27]. An expert scientist checked each patient’s STAS condition.

### 2.3. Ethical Statement of Ethical Approval for the Study

The institutional ethics committee of Health Sciences University Affiliated Kartal Doctor Lütfi Kırdar City Hospital authorized it on 29 November 2023, with decision number 2023/514/262/12. The procedures used for retrospective data collection, patient file review, and study conduct were conducted in accordance with the ethical standards set by the institutional and/or national research committee and were also aligned with the 1964 Declaration of Helsinki and its subsequent amendments.

### 2.4. Statistical Analysis

Statistical analyses were performed using IBM SPSS Statistics for Windows, Version 25.0 (Statistical Package for the Social Sciences, IBM Corp., Armonk, NY, USA). The descriptive statistics included the median (range) for continuous variables and the count and percentage for categorical variables. DFS was defined as the time interval between the surgical procedure and the occurrence of disease reoccurrence, death, or the final visit. OS was defined as the time from diagnosis to either death from any cause or the last recorded visit. The Kaplan–Meier technique and log-rank test were used to conduct survival analysis. The impact of prognostic variables on survival was assessed using the univariate log-rank test. The hazard ratio (HR) was computed using a 95% confidence interval (CI). The Cox proportional hazards model was used to conduct a multivariate analysis in order to evaluate the impact of prognostic variables on survival. The significance threshold was determined to be less than or equal to 0.05.

## 3. Results

### 3.1. Patient Characteristics

Follow-up and treatment were performed at Kartal Doctor Lütif Kırdar City Hospital Medical Oncology Clinic, and 311 patients who met the inclusion criteria and not the exclusion criteria were included in the study. In total, 63 (20%) of the patients were female, and 248 (80%) were male. The median age of the patients was 63 years (min 37–max 82). While 44 (14%) patients had never smoked, 260 (84%) patients had smoked or were still smoking. A total of 142 (46%) patients had squamous cell carcinoma, 169 (44%) patients had adenocarcinoma, and 33 (10%) had other lung cancer subtypes. In total, 111 (35.6%) patients had stage 1A1–1B, 112 (36%) patients had stage 2A–2B, 75 (24.1%) patients had stage 3A, and 13 (4%) patients had stage ≥ 3B disease. The median follow-up period was 40 (min 4–max 86) months. Adjuvant chemotherapy was administered to 217 (70%) patients, while 94 (30%) patients were followed without adjuvant chemotherapy. The distribution of patients’ sociodemographic and clinicopathological characteristics of the tumors is shown in Table 1.

### 3.2. Association between Clinical Factors and STAS

A total of 103 (33%) patients were STAS-positive, and 208 (67%) were STAS-negative. STAS positivity was significantly more common in the adenocarcinoma subtype (*p* = 0.01). Perineural invasion (PNI) and lymphovascular invasion (LVI) were significantly more frequent in STAS-positive patients than in STAS-negative patients (*p* = 0.009 and *p* = 0.003, respectively). Additionally, STAS positivity increased significantly with increasing lymph node stage (*p* = 0.04). The distribution of patients according to STAS status is shown in Table 2.

### 3.3. Survival Outcomes

In a median follow-up of 40 months (range 4–86 months), 137 (44%) patients experienced recurrence, and 79 (25%) patients died. The median DFS and OS for the entire group are shown in Table 1.

Univariate survival analysis for DFS in the entire patient group showed that PET-CT SUVmax (*p* = 0.02), pathological stage (*p* = 0.006), and STAS (*p* < 0.01) were significant risk factors for DFS, while multivariate survival analysis only showed PET-CT SUVmax (*p* = 0.01) and STAS status (*p* < 0.001) to be statistically significant (Table 3).

Similarly, univariate survival analysis for OS in the entire patient group showed that PET-CT SUVmax (*p* = 0.02), pathological stage (*p* = 0.002), lymphovascular invasion (*p* = 0.030), and STAS (*p* < 0.01) were prognostic factors for OS, while multivariate survival analysis only showed pathological stage (*p* = 0.005) and STAS status (*p* < 0.001) to be statistically significant (Table 4).

#### 3.3.1. Relationship between DFS and STAS

When patients were evaluated according to their STAS status, the recurrence rate was 65% (67/103) in the STAS-positive group and 34% (70/208) in the STAS-negative group (*p* < 0.001). Median DFS was 22 months (95%CI: 13.8–30.1) in the STAS-positive group, while the median value was not reached in the STAS-negative group (*p* < 0.001).

**Figure 1:**
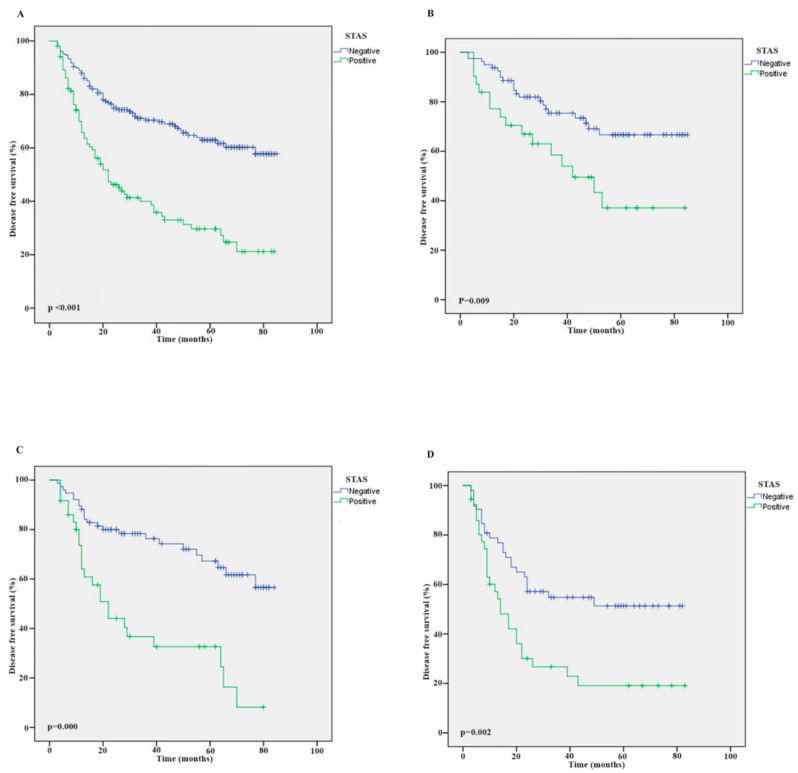
Disease-free survival outcomes by Kaplan–Meier graphic according to STAS status. (**A**) Whole cohort. (**B**) Stage 1 patient group according to STAS status. (**C**) Stage 2 patient group according to STAS status. (**D**) Stage 3 patient group according to STAS status. STAS: spread through air spaces.

In both the entire patient group and pathological Stage 1, pathological Stage 2, and pathological Stage 3 groups, separately, DFS was lower in the STAS-positive group than in the STAS-negative group, regardless of the stage (*p* < 0.001, *p* = 0.009, *p* < 0.000, and *p* = 0.002, respectively). Figure 1 shows the survival results of the Kaplan–Meier analysis for the entire patient group and by pathological stage.

#### 3.3.2. Relationship between OS and STAS

The mortality rate was 38% (39/103) in the STAS-positive group and 19% (40/208) in the STAS-negative group (*p* < 0.001) when evaluated by STAS status. The median OS was 66 months (95%CI: 42.1–89.8) in the STAS-positive group and was not reached in the STAS-negative group (*p* < 0.001).

**Figure 2:**
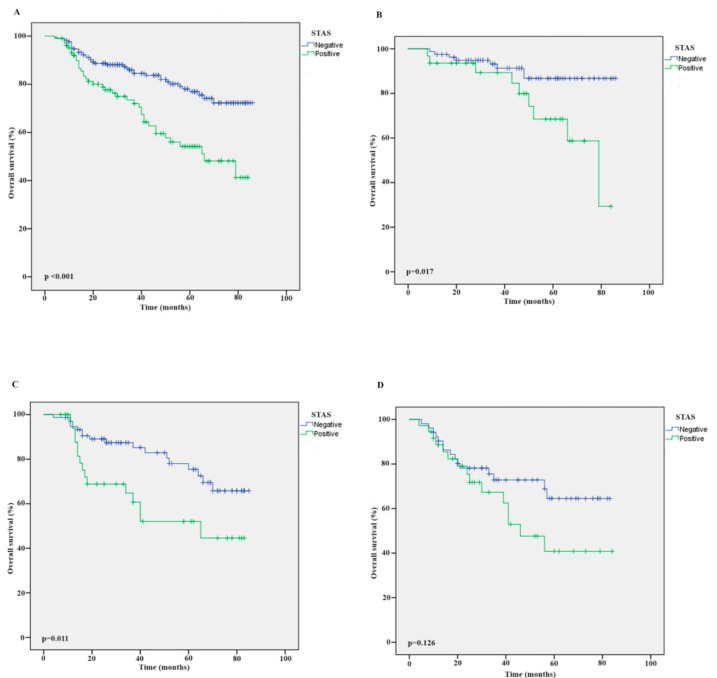
Overall survival outcomes by Kaplan–Meier graphic according to STAS status. (**A**) Whole cohort. (**B**) Stage 1 patient group according to STAS status. (**C**) Stage 2 patient group according to STAS status. (**D**) Stage 3 patient group according to STAS status. STAS: spread through air spaces.

In both the entire patient group and pathological Stage 1 and pathological Stage 2 groups, separately, STAS-positive patients had significantly lower OS than STAS-negative patients (*p* < 0.001, *p* = 0.017 and *p* < 0.011, respectively). Although STAS-positive patients in the pathological Stage 3 group had a lower OS than those who were STAS-negative, the difference was not statistically significant (*p* = 0.126). Figure 2 shows the survival results of the Kaplan–Meier analysis for all patients and by pathological stage. The DFS and OS results according to STAS status are shown in Table 5.

## 4. Discussion

In current clinical practice, surgical resection and appropriate neoadjuvant or adjuvant therapy remain the standard for early-stage lung cancer [5]. However, a significant number of patients experience recurrence, which can lead to a significant decrease in OS and quality of life. Many clinicopathological features can predict recurrence. There is mounting evidence that indicates the importance of STAS in predicting the prognosis of lung cancer [28]. Our study aimed to demonstrate the effect of STAS, which is gaining more importance with each passing day, on survival and recurrence in patients with early-stage resected NSCLC.

After Kadota et al. described STAS in 2015, numerous studies have been conducted on its clinicopathological and molecular characteristics and prognostic significance [28,29,30]. It has been discussed that STAS positivity may influence the surgical procedure to be applied. Studies have shown that patients with STAS positivity who undergo limited resection have worse prognoses than those undergoing lobectomy [31]. However, STAS has insufficient representation in daily practice and treatment guidelines [32].

Clinical studies have shown that STAS-positive patients exhibit certain clinicopathological features that differ from STAS-negative patients [28,29,30,31,32,33]. In a study by Chen et al., histological grade, histological type, and vascular invasion were significantly associated with STAS [28]. Another study found that STAS presence was related to advanced N stage, poorly differentiated histological grade, pleural invasion, and lymphovascular invasion [30]. Furthermore, studies have demonstrated significant associations between STAS presence and morphological patterns, maximum tumor diameter, vascular invasion, neural invasion, lymph node metastasis, and clinical stage [32,33]. Our study also observed significant associations between STAS presence and histological type, N stage, pleural invasion, perineural invasion, and lymphovascular invasion.

While some strong studies suggest that STAS leads to adverse clinical outcomes in OS and DFS, others show the opposite [34,35]. In a study by Wang et al., STAS presence was included among the pathological features that adversely affect OS and progression-free survival (PFS) in stage 1 patients [36]. Another similarly designed study revealed STAS positivity as an independent prognostic factor for unfavorable DFS and OS [28]. In a study on stage 1 patients, it was reported that STAS positivity had a negative impact on RFS and OS and that patients with stage 1B and STAS positivity benefited from adjuvant chemotherapy in terms of prolonged RFS [37]. Our study confirmed that STAS presence is an adverse prognostic factor for DFS and OS in stage 1 patients. No prior research specifically evaluated stage 2 patients specifically; however, our study demonstrated that STAS positivity significantly lowers DFS and OS in stage 2 patients. Likewise, no studies currently exist specifically addressing stage 3 patients; our study showed a significant relationship between STAS positivity and lower DFS, with a trend toward lower OS that did not reach statistical significance.

A meta-analysis that included 47 studies concluded that STAS presence shows a significant correlation with aggressive tumor behavior and poor prognosis [32]. Another meta-analysis of approximately 3750 patients demonstrated a significant relationship between STAS presence and unfavorable DFS and OS. Subgroup analysis by histological type found STAS presence significantly associated with lower DFS in resected lung adenocarcinoma and lung squamous cell carcinoma [38]. In a study on patients with stage I-III resected lung adenocarcinoma, STAS presence was associated with lower PFS and OS [39]. Another study of patients with Stage I-III lung squamous cell carcinoma found a significant association between STAS presence and higher rates of recurrence and lung cancer-related death [19].

As the importance of STAS becomes clearer, it is stated that it will be understood to be an important prognostic factor such as visceral pleural invasion (VPI) and LVI in the staging study of lung cancer conducted by The International Association for the Study of Lung Cancer (IASLC) with the further maturation of information in the coming years. The research also indicates that STAS might be included in the 10th TNM classification of lung cancer [40]. Studies have been conducted to develop a prediction model for the presence of STAS in early-stage NSCLC using imaging and genetic characteristics of STAS, recognizing its significance [17,41]. Studies have been conducted to develop a prediction model for precise the intraoperative diagnosis of STAS in early-stage NSCLC. This model is based on the analysis of imaging and genetic characteristics of STAS, recognizing the significance of STAS [24,42].

Our study has some limitations, including its single-center and retrospective nature. Nevertheless, its strengths include evaluating survival for an entire patient group simultaneously across stages 1, 2, and 3.

## 5. Conclusions

STAS is an aggressive pathological feature detected in a significant proportion of lung cancer patients. There is a significant association between STAS presence and reduced DFS and OS; it serves as a negative independent prognostic factor. STAS, frequently present alongside negative pathological features such as high grade and vascular invasion, is a poor prognostic factor for resected stage 1, 2, and 3 lung cancer. Including STAS status in pathology reports is essential for guiding clinicians in determining appropriate treatment approaches for potentially improved prognostic outcomes. However, our study should be supported by additional multicenter and prospective studies.

## Figures and Tables

**Table 1:** Baseline clinical and demographic findings of whole cohort.

**Variables**		n = 311 (%)
**Gender**	Female	63 (20)
	Male	248 (80)
Age (median)	62 years (min 37–max 82)	
BMI (median) kg/m^2^	26.1 (min 17.5–max 45.9)	
Smoking status	Never Smoker	44 (14)
	Active/Ex Smoker	260 (84)
	Unknown	7 (2)
ECOG PS	PS 0	185 (60)
	PS 1	112 (36)
	PS ≥ 2	14 (4)
PET-CT (median)	Tumor diameter (cm)	3.2 (min 0.6–max 10.5)
	Tumor Suv max	10.6 (min 2–max 47)
Neoadjuvan chemotherapy	Present	30 (10)
	Absent	281 (90)
Pathologic Subtype	Adenocarcinoma	136 (44%)
	Squamous cell carcinoma	142 (46%)
	Other	33 (10%)
Mutation Status (n = 122)	Undetected (no mutation)	108 (88.5)
	EGFR mutant	12 (9.8)
	Braf mutant	1 (0.8)
	Ros-1 mutant	1 (0.8)
Pathologic Stage	1A1	18 (6)
	1A2	40 (14)
	1A3	21 (7)
	1B	32 (10)
	2A	30 (9)
	2B	82 (26)
	3A	75 (24)
	≥3B	13 (4)
Lympovascular Invasion	Present	109 (35)
	Absent	202 (65)
Perineural Invasion	Present	72 (21)
	Absent	239 (79)
Pleural Invasion	Present	98 (32)
	Absent	213 (68)
STAS	Present	103 (33)
	Absent	208 (67)
Adjuvan Chemotherapy	Yes	217 (70)
	No	94 (30)
Adjuvan Radiotherapy	Yes	38 (12)
	No	273 (88)
Recurrence Status	Present	137 (44)
	Absent	174 (56)
Disease Free Survival	Median (months)	65 (95%CI)
	1 years (%)	82
	5 years (%)	52
Status	Exitus	79 (25)
	Alive	232 (75)
Overall Survival	Median (Months)	Not reached
	1 years (%)	93
	5 years (%)	69

BMI: Body Mass Index, ECOG PS: Eastern Cooperative Oncology Group performance status, PET-CT: positron emission tomography-computed tomography scan, STAS: spread through air spaces.

**Table 2:** Demographic and clinical findings according to STAS status.

Variables		STAS Status		
		Present (n = 103)	Absent (n = 208)	*p*
**Gender**	Female	20 (19%)	43 (21%)	0.79
	Male	83 (81%)	165 (79%)	
Age (years) median		62 (min 46–max 82)	62 (min 37–max 82)	0.67
Smoking Status	Never smoker	17 (16%)	27 (13%)	0.41
	Active-ex smoker	85 (84%)	175 (87%)	
PET-CT SUV max (median)		11.3 (min 3–max 47)	10.4 (min 2–max 40)	0.52
Resection type	Wedge resection	4 (3%)	18 (9%)	0.34
	Lobectomy	82 (81%)	155 (76%)	
	Bilobectomy	6 (5%)	8 (2%)	
	Pneumonectomy	11 (11%)	27 (13%)	
Pathologic Subtype	Adenocarcinoma	54 (52%)	82 (39%)	**0.01**
	Squamous cell carcinoma	34 (33%)	108 (52%)	
	Other	15 (15%)	18 (8%)	
Adenocarcinoma subtype (n = 117)	Lepidic	10 (22%)	13 (18%)	0.18
	Solid	14 (30%)	21 (30%)	
	Acinary	19 (41%)	28 (39%)	
	Micropapillary	3 (6%)	1 (2%)	
	Papillary	0	6 (8%)	
	Mucinous	0	2 (3%)	
PD-L1 score (median) (n = 24)		6 (min 0–max 90)	1 (min 0–max 90)	0.36
Pathologic tumor diameter (median) (cm)		3.1 (min 0.6–max 15)	3.5 (min 0.2–max 3.5)	0.73
Pathologic T stage	T1	34 (33%)	79 (38%)	0.63
	T2	36 (35%)	63 (30%)	
	T3	18 (17%)	42 (20%)	
	T4	15 (15%)	24 (12%)	
Pathologic N stage	N0	57 (55%)	137 (65%)	**0.04**
	N1	28 (27%)	53 (25%)	
	N2	18 (18%)	18 (10%)	
Pathologic stage	Stage 1	31 (30%)	80 (38%)	0.14
	Stage 2	36 (35%)	76 (37%)	
	Stage 3	36 (35%)	52 (25%)	
Lympovascular Invasion	Present	48 (47%)	61 (29%)	**0.003**
	Absent	55 (53%)	147 (71%)	
Perineural Invasion	Present	33 (32%)	39 (19%)	**0.009**
	Absent	70 (68%)	169 (81%)	
Pleural Invasion	Present	40 (39%)	58 (28%)	**0.05**
	Absent	63 (61%)	150 (72%)	
Adjuvan Chemotherapy	Yes	76 (74%)	141 (68%)	0.27
	No	27 (36%)	67 (32%)	

STAS: spread through air spaces, PET-CT SUV: positron emission tomography-computed tomography scan standardized uptake value, PD-L1: Programmed Death-Ligand 1.

**Table 3:** Cox regression model for disease-free survival (DFS) in the whole cohort.

		Disease-Free Survival			
		Univariate Analysis		Multivariate Analysis	
		HR (95%CI)	*p*	HR (95%CI)	*p*
**Age**	<65 years	1 (0.7–1.4)	0.980		
	≥65 years				
Gender	Female	1 (0.8–1.3)	0.390		
	Male				
Pet-ct Suv max	≥10	1.6 (1–2.5)	**0.020**	1.7 (1.1–2.6)	**0.010**
	<10				
Pathologic subtype	Adenocarcinoma	0.94 (0.6–1.3)	0.760		
	Squamous cell carcinoma				
Pathologic stage	Stage 1	1.68 (1.1–2.4)	**0.006**		
	Stage 2–3				
Lympovascular Invasion	Present	1.3 (0.96–1.9)	0.070		
	Absent				
Perineural Invasion	Present	1.1 (0.82–1.7)	0.340		
	Absent				
Pleural Invasion	Present	1.2 (0.8–1.7)	0.280		
	Absent				
STAS	Present	2.7 (1.98–3.8)	<0.010	3.2 (2.1–4.8)	**<0.001**
	Absent				

Pet-ct Suv: positron emission tomography-computed tomography scan standardized uptake value, STAS: spread through air spaces.

**Table 4:** Cox regression model for overall survival (OS) in the whole cohort.

		Overall Survival			
		Univariate Analysis		Multivariate Analysis	
		HR (95%CI)	*p*	HR (95%CI)	*p*
**Age**	<65 years	1.1 (0.7–1.7)	0.630		
	≥65 years				
Gender	Female	1.3 (0.9–1.8)	0.080		
	Male				
Pet-ct Suv max	≥10	2.06 (1.1–3.8)	**0.020**		
	<10				
Pathologic subtype	Adenocarcinoma	1.1 (0.7–1.7)	0.670		
	Squamous cell carcinoma				
Pathologic stage	Stage 1	2.3 (1.3–4.02)	**0.002**	2.8 (1.3–5.4)	**0.005**
	Stage 2–3				
Lympovascular Invasion	Present	1.7 (1.1–2.7)	**0.030**		
	Absent				
Perineural Invasion	Present	1.2 (0.7–1.9)	0.440		
	Absent				
Pleural Invasion	Present	1.1 (0.7–1.8)	0.460		
	Absent				
STAS	Present	2.3 (0.7–3.64)	<0.010	3.1 (1.7–5.5)	**<0.001**
	Absent				

Pet-ct Suv: positron emission tomography-computed tomography scan standardized uptake value, STAS: spread through air spaces.

**Table 5:** Survival outcomes according to STAS status.

		STAS		
		Present (n = 103)	Absent (n = 208)	*p*
Recurrence Status	Present	67 (65%)	70 (34%)	**<0.001**
	Absent	36 (35%)	138 (66%)	
Disease-Free Survival	Median (months)	22 (95%CI: 13.8–30.1)	Not reached	**<0.001**
	1 years (%)	65	88	
	5 years (%)	29	62	
Overall Status	Exitus	39 (38%)	40 (19%)	**<0.001**
	Alive	64 (62%)	168 (81%)	
Overall Survival	Median (Months)	66 (95%CI: 42.1–89.8)	Not reached	**<0.001**
	1 years (%)	92	94	
	5 years (%)	54	88	

STAS: spread through air spaces.

## Data Availability

Data are contained within the article.
